# Prevalence and risk factors for chronic kidney disease of unknown cause in Malawi: a cross-sectional analysis in a rural and urban population

**DOI:** 10.1186/s12882-020-02034-x

**Published:** 2020-09-07

**Authors:** Sophie A. Hamilton, Wisdom P. Nakanga, Josephine E. Prynn, Amelia C. Crampin, Daniela Fecht, Paolo Vineis, Ben Caplin, Neil Pearce, Moffat J. Nyirenda

**Affiliations:** 1 Department of Epidemiology and Biostatistics, Imperial College London, London, UK; 2 MRC Centre for Environment and Health, Imperial College London, London, UK; 3 Imperial College London, School of Public Health, London, UK; 4 Malawi Epidemiology and Intervention Research Unit, Lilongwe, Malawi; 5 Institute of Cardiovascular Science, University College London, London, UK; 6 Departments of Infectious Disease Epidemiology, London School of Hygiene and Tropical Medicine, London, UK; 7 Centre for Nephrology, Division of Medicine, University College London, London, UK; 8 Department of Medical Statistics, London School of Hygiene and Tropical Medicine, London, UK; 9 Centre for Global NCDs, London School of Hygiene and Tropical Medicine, London, UK

**Keywords:** Epidemiology, Chronic kidney disease, Estimated glomerular filtration rate, Prevalence, Risk factor, Sub-Saharan Africa

## Abstract

**Background:**

An epidemic of chronic kidney disease of unknown cause (CKDu) is occurring in rural communities in tropical regions of low-and middle-income countries in South America and India. Little information is available from Southern African countries which have similar climatic and occupational characteristics to CKDu-endemic countries. We investigated whether CKDu is prevalent in Malawi and identified its potential risk factors in this setting.

**Methods:**

We conducted a cross-sectional study from January–August 2018 collecting bio samples and anthropometric data in two Malawian populations. The sample comprised adults > 18 years (*n* = 821) without diabetes, hypertension, and proteinuria. Estimates of glomerular filtration rate (eGFR) were calculated using the CKD-EPI equation. Linear and logistic regression models were applied with potential risk factors, to estimate risk of reduced eGFR.

**Results:**

The mean eGFR was 117.1 ± 16.0 ml/min per 1.73m^2^ and the mean participant age was 33.5 ± 12.7 years. The prevalence of eGFR< 60 was 0.2% (95% confidence interval (95% CI) 0.1, 0.9); the prevalence of eGFR< 90 was 5% (95% CI =3.2, 6.3). We observed a higher prevalence in the rural population (5% (3.6, 7.8)), versus urban (3% (1.4, 6.7)). Age and BMI were associated with reduced eGFR< 90 [Odds ratio (OR) (95%CI) =3.59 (2.58, 5.21) per ten-year increment]; [OR (95%CI) =2.01 (1.27, 3.43) per 5 kg/m^2^ increment] respectively. No increased risk of eGFR < 90 was observed for rural participants [OR (95%CI) =1.75 (0.50, 6.30)].

**Conclusions:**

Reduced kidney function consistent with the definition of CKDu is not common in the areas of Malawi sampled, compared to that observed in other tropical or sub-tropical countries in Central America and South Asia. Reduced eGFR< 90 was related to age, BMI, and was more common in rural areas. These findings are important as they contradict some current hypothesis that CKDu is endemic across tropical and sub-tropical countries. This study has enabled standardized comparisons of impaired kidney function between and within tropical/subtropical regions of the world and will help form the basis for further etiological research, surveillance strategies, and the implementation and evaluation of interventions.

## Background

An epidemic of chronic kidney disease of unknown cause (CKDu) is occurring in rural communities in an increasing number of low-and middle-income countries (LMICs) [1, 2]. The highest prevalence of CKDu has been reported in Nicaragua, Central America [3, 4] where 10–20% of the adult population are affected [5, 6]. CKDu has also been reported in other rural communities across Southern India and Sri Lanka where the prevalence is 1.6 and 1.5% respectively [7, 8].

CKDu is defined by an estimated glomerular filtration rate (eGFR) of < 60 ml/min per 1.73m^2^ in the absence of traditional risk factors such as diabetes, hypertension, heavy proteinuria, and structural renal disease, based on two measures of serum creatinine 3 months apart [2, 9, 10]. Histopathology reports from previous CKDu studies show that tubular atrophy and interstitial fibrosis in the presence of varying degrees of glomerulosclerosis are the predominant features of CKDu [2, 11]. However, to date, there are few examples of consistent approaches and case definitions across countries which have conducted CKDu research, which makes international comparisons challenging [2, 12]. As a result, it is unclear whether there is an underlying aetiology or causal pathway of the condition, and indeed whether it occurs in other regions of the world. However, there is consensus in the research landscape on the possible range of risk factors – predominantly heat stress [13–15], agricultural labour [16] and heavy metal exposure [17], which are factors largely associated with rural habitation.

CKDu has been reported in Central America and South Asia, however little information is available from Southern African countries, which have similar climatic and occupational characteristics to reported CKDu-endemic regions. Malawi was selected as our study location as previous research in urban and rural Malawi demonstrated a significant association with impaired kidney function and agricultural labour – a central hypothesis in the CKDu literature [18] which warranted further research measuring eGFR applying a different diagnostic definition.

The aim of this study was to estimate the distribution of eGFR in an urban and rural area of Malawi to identify the presence of CKDu. We aimed to: (i) assess eGFR distribution and prevalence of eGFR below 60 ml/min per 1.73m^2^ (eGFR< 60) and eGFR below 90 ml/min per 1.73m^2^ (eGFR < 90) in a population restricted to those without known risk factors for CKD, i.e. diabetes, hypertension or heavy proteinuria; (ii) compare these outcomes between urban and rural populations; and (iii) identify anthropometric and lifestyle-related risk factors associated with these outcomes.

## Methods

The Disadvantaged Populations eGFR Epidemiology (DEGREE) Study [1] is a collaboration which has produced a standardised protocol for estimating the population distribution of the estimated glomerular filtration rate (eGFR) distribution in LMICs. This involves quantification of renal function in a representative adult population-based sample with standardisation of serum creatinine measurements, storage of samples for future measurements of cystatin C and ascertainment of body composition estimates to obtain valid comparisons within and between populations. The DEGREE methodologies are described in detail elsewhere [1]. Here we report the results from a DEGREE study conducted in Malawi, south-east Africa.

### Study population

**Fig. 1:**
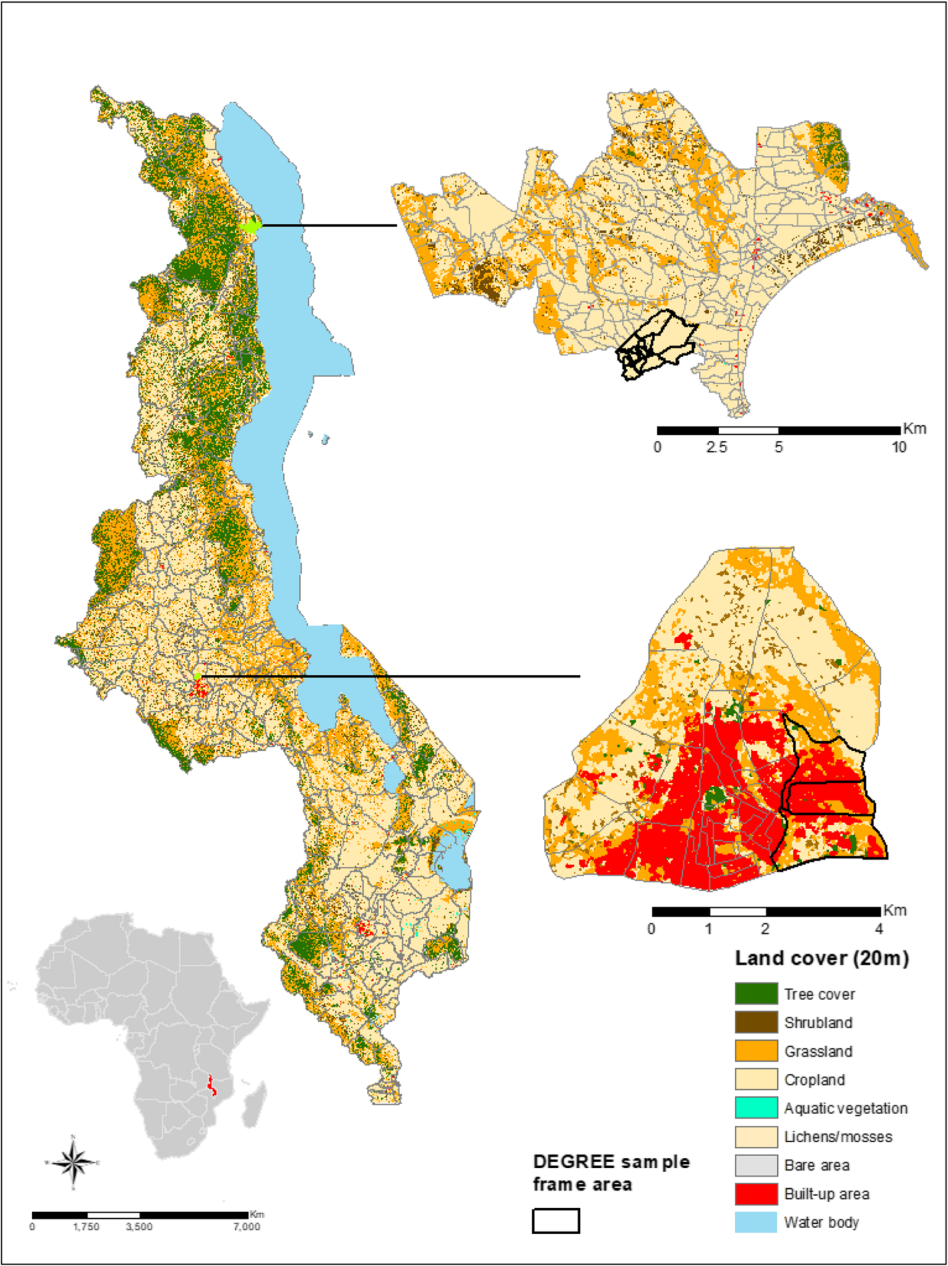
Malawi study sites; a) Bonje sample frame; b) Area 25 sample frame

The DEGREE survey was conducted in northern rural Karonga District, and Malawi’s capital city Lilongwe (Fig. 1). The rural survey in Karonga District was nested in the well-established Health and Demographic Surveillance Site (HDSS) (135km^2^ area, population 40,000) [19] in a predominantly subsistence economy. The sample frame is located in Bonje, a village area including a large commercial rice irrigation scheme. The urban survey in Lilongwe was situated in a sub-area of Area 25 (10km^2^ area, population 66,000 [20]) a high-density, economically mixed residential area. The sub-area selected is one of the more deprived areas where casual and seasonal labourers reside.

In Karonga, 1200 participants aged 18 years and older were randomly sampled at the individual level from the existing HDSS sample frame for the selected area. In Lilongwe, it was anticipated that many residents would have moved since the 2013 enumeration for the NCD survey. We re-enumerated the area and used a new list of residents as a sampling frame. This was done by dividing the geographical area within Area 25 into zones of approximately 100 households each and enumerating each at a time. After each zone was enumerated, 1 in 3 adults were randomly sampled.

### Data collection and laboratory analyses

Data collection was conducted between January and August 2018. Participants were interviewed using a standardized questionnaire collecting data on basic co-variates (age, sex, years of schooling), lifestyle factors (alcohol intake, smoking, meat consumption as number of days per week), medical history (previous diagnoses of hypertension, diabetes and analgesic use), and occupational status (including information on workload and financial income).

Height and weight were measured twice per visit for each participant using calibrated Stadiometer seca-213, Scales seca-761 apparatus respectively (seca GmbH & Co. KG, Germany), and the mean values calculated. Body composition was measured using a Bodystat 1500 bioimpedance analysis (BIA) instrument (Bodystat Ltd., United Kingdom). Fat-free mass (FFM) was calculated using the mean value of two bioimpedance output measures (fat free mass/lean mass, kg). Body mass index (BMI, kg/m^2^) was calculated and categorized as underweight: ≤18.5, normal weight: > 18.5- ≤ 25, overweight: > 25- ≤ 30, and obese: > 30.

Blood pressure was measured with a portable electronic device OMRON HEM-7211-E, Model M6 (OMRON Healthcare Co., Ltd., Japan) on the right upper arm, three times in a seated position after 30 min of inactivity and 5 min rest between measurements. Participants were classified as having hypertension if systolic blood pressure was ≥140 mmHg, or diastolic blood pressure was ≥90 mmHg, or if the participant was on antihypertensive medication.

First-urine (early morning) and fasting venous blood samples were taken by clinical field staff. Urine was tested for albumin using the photometric colour test method, and blood samples were tested using the hexokinase and Kinetic Jaffé compensated methods to measure glucose and serum creatinine. Serum creatinine samples were measured in the Malawi Epidemiology and Intervention Research Unit laboratories located in Lilongwe and Karonga using a method calibrated to isotope dilution mass spectrometry (IDMS) standards. Participants were categorized as heavily proteinuric if the albumin: creatinine ratio (ACR) was ≥30 mg/mmol, and diabetic if fasting plasma glucose was ≥7 mmol/L, or if the participant self-reported diabetes and on hypoglycaemic medication.

The CKD Epidemiology Collaboration (CKD-EPI) equation was used to calculate eGFR [1]. No correction for ethnicity was applied as many studies from Sub Saharan Africa which include coefficients for African American ethnicity consistently overestimate GFR [21–25]. Clinical diagnosis of CKDu is based on two measures 3 months apart [26], which is not practical in most epidemiological studies, thus our main outcome measure was eGFR< 60 without known risk factors of kidney disease [7].

### Statistical analysis

The DEGREE protocol specifies a diagnostic cut off for impaired kidney function of eGFR< 60 ml/min per 1.73m^2^ for reduced function in the absence of diabetes, hypertension, and heavy proteinuria [1]. There were few cases of impaired kidney function using this definition (see Results below). We therefore decided to investigate risk factors for an eGFR below normal (< 90 ml/min per 1.73m^2^), in the absence of known CKD risk factors.

We used linear regression models to estimate associations between potential risk factors (Table 1) and continuous eGFR, and logistic regression to estimate associations between risk factors and reduced eGFR.

**Table 1:** Sociodemographic and anthropometric characteristics of study participants without diabetes, hypertension, and heavy proteinuria) *n* = 821

Variable	eGFR		eGFR categories, n(%)^b^		
	*n* = 821		*n* = 2	*n* = 36	*n* = 783
	n(%)^a^	Mean (SD)	< 60	> = 60, < 90	> = 90
Age (years)					
< 20	60 (7)	132.6 (13.2)	0	1 (2)	59 (98)
20–29	312 (38)	125.1 (12.6)	0	2 (1)	310 (99)
30–39	232 (28)	115.7 (12.5)	1 (0.4)	4 (2)	227 (98)
40–49	130 (16)	107.8 (11.1)	1 (1)	2 (2)	127 (98)
50–59	46 (6)	100.9 (8.6)	0	6 (12)	40 (87)
60+	41 (5)	88.9 (9.9)	0	21 (51)	20 (49)
Sex					
Female	504 (61)	117.3 (15.6)	2 (0.4)	20 (4)	482 (96)
Male	317 (39)	116.81 (16.7)	0	16 (5)	301 (95)
Area					
Urban (Area 25)	243 (29)	117.6 (14.9)	1 (0.4)	6 (3)	236 (97)
Rural (Bonje)	578 (71)	116.9 (16.5)	1 (0.2)	30 (5)	547 (95)
Education (years)					
≤ 5	59 (7)	109.1 (17.9)	0	9 (15)	50 (85)
> 5 ≤ 10	301 (36)	116.6 (16.4)	1 (0.2)	15 (3)	285 (62)
> 10	461 (56)	118.5 (15.2)	1 (0.3)	12 (4)	448 (14)
Occupation					
Agricultural worker	383 (47)	113.9 (15.2)	1 (0.3)	20 (5)	362 (95)
Non-agricultural worker	438 (53)	119.90 (16.2)	1 (0.2)	16 (4)	421 (96)
Household monthly income (MK)^c^					
Unknown	17 (1)	118.6 (21.1)	1 (6)	0	16 (94)
MK 0 ≤ 20,000	406 (50)	117.8 (16.6)	0	20 (5)	386 (95)
MK > 20,000	398 (49)	116.3 (15.1)	1 (0.3)	16 (4)	381 (96)
Healthy lifestyle choices					
Non-smoker/ never drink alcohol	630 (77)	117.0 (15.8)	2 (0.3)	28 (4)	600 (95)
Smoker/ alcohol drinker	191 (23)	117.3 (16.4)	0	8 (4)	183 (96)
Regular meat-eater					
Yes	621 (76)	117.5 (15.6)	2 (0.3)	25 (4)	594 (96)
No	200 (24)	115.6 (16.1)	0	11 (6)	189 (95)
Body mass index (kg/m^2^)					
Underweight (≤18.5)	45 (6)	121.6 (18.0)	0	3 (7)	42 (93)
Normal (> 18.5 - ≤ 25)	545 (66)	118.4 (15.4)	0	17 (3)	528 (97)
Overweight (> 25 - ≤30)	177 (22)	113.3 (15.1)	2 (1)	7 (4)	168 (95)
Obese (> 30)	54 (7)	112.1 (17.8)	0	9 (17)	45 (83)
Fat-free mass (kg)					
1st tertile (≤37)	124 (15)	115.1 (16.3)	0	8 (7)	116 (94)
2nd tertile (> 37 - < 45)	354 (43)	116.9 (16.9)	2 (1)	18 (5)	334 (94)
3rd tertile (≥45)	343 (42)	117.9 (14.9)	0	10 (3)	333 (97)
HIV status					
Positive	3 (0.4)	114.3 (15.1)	0	0	3 (100)
Negative	595 (73)	116.8 (15.1)	1 (0.2)	24 (4)	570 (96)
Unknown	223 (27)	118.0 (18.1)	1 (0.4)	12 (5)	210 (94)

^a^percentage in columns

^b^percentage in rows

^c^Exchange rate (MK to USD) 0.001 at time of questionnaire

We repeated analyses separately for urban and rural areas. We re-grouped co-variates into broader categories before conducting analyses (See Additional file 1, Table S1 for original categories) due to the small proportion of those with eGFR< 90.

Variables associated with lower eGFR in univariate analyses (adjusted for age, sex, and study site) were included in the multiple regression analysis. In the final multiple regression model, variables showing independent association with eGFR, and those of a-priori interest identified via literature searches (study site, sex, and education) were included.

We checked for multicollinearity of each variable in the multiple regression analysis in comparison to the univariate analysis [27]. All analyses were conducted using R studio version 3.5.1.

## Results

### Study population characteristics

**Fig. 2:**
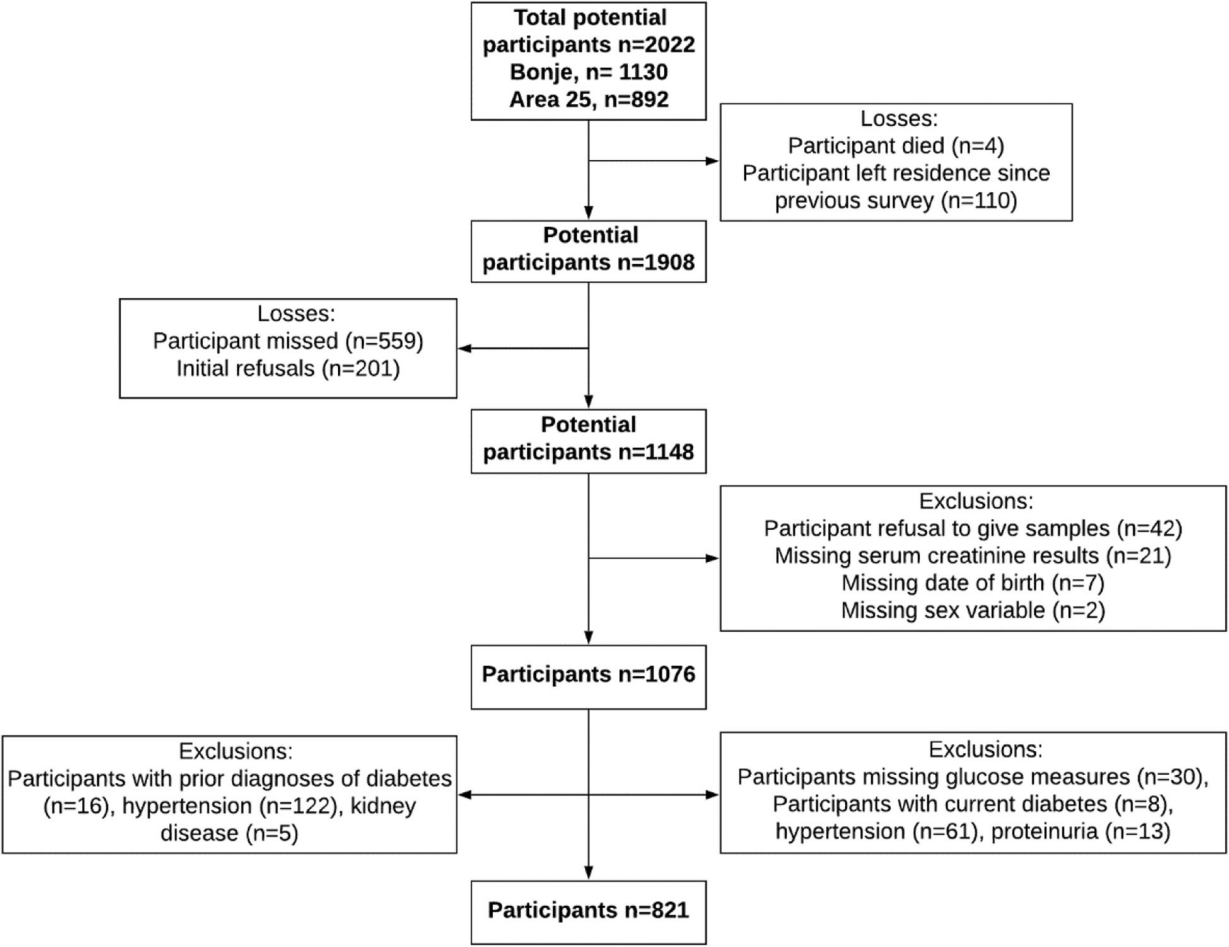
Flow diagram of participants excluded for analyses

The overall response rate was 56% (*n* = 1076 participants out of 1908 eligible; 654 females, 422 males). Participants were “missed” if field staff had visited the residence on three occasions to invite them to participate in the study but did not find them at home (*n* = 559; 189 females, 367 males, 3 missing all data variables). A total of 201 individuals were found but were unwilling to participate in the informed consent procedures (initial refusers) (115 females, 83 males, 3 missing all data variables). Using a priori exclusion criteria, we then removed participants with missing basic variables including date of birth, and those with the known CKD risk factors diabetes, hypertension, or heavy proteinuria (Fig. 2). After exclusions, 821 participants were eligible for analysis (Fig. 2). Table 1 summarizes socio-demographic and anthropometric characteristics of the sample (see Additional file 1, Table S1 for the non-restricted sample, *n* = 1076).

The mean (standard deviation (±SD)) participant age was 33.5 ± 12.7 years. Mean BMI was 23.4 ± 3.9 kg/m^2^, and mean FFM was 51.7 ± 82.3 kg. Mean systolic and diastolic blood pressure were 116.5 ± 10.3 mmHg, and 70.7 ± 8.3 mmHg, respectively. Mean fasting plasma glucose was 4.7 ± 0.7 mmol/l and the median (interquartile range IQR) albumin: creatinine ratio (ACR) was 0.5 (0.8) mg/mmol after exclusion of those with ACR > =30 mg/mmol, *n* = 13).

There was an 82% employment rate in the population (females, *n* = 390 [77%], males, *n* = 286 [90%]), approximately 57% of which were classified as agricultural workers (females, *n* = 235 [61%], males, *n* = 148 [39%]). 56% of the population completed ≥10 years of formal education.

### Mean eGFR and prevalence of reduced eGFR

**Fig. 3:**
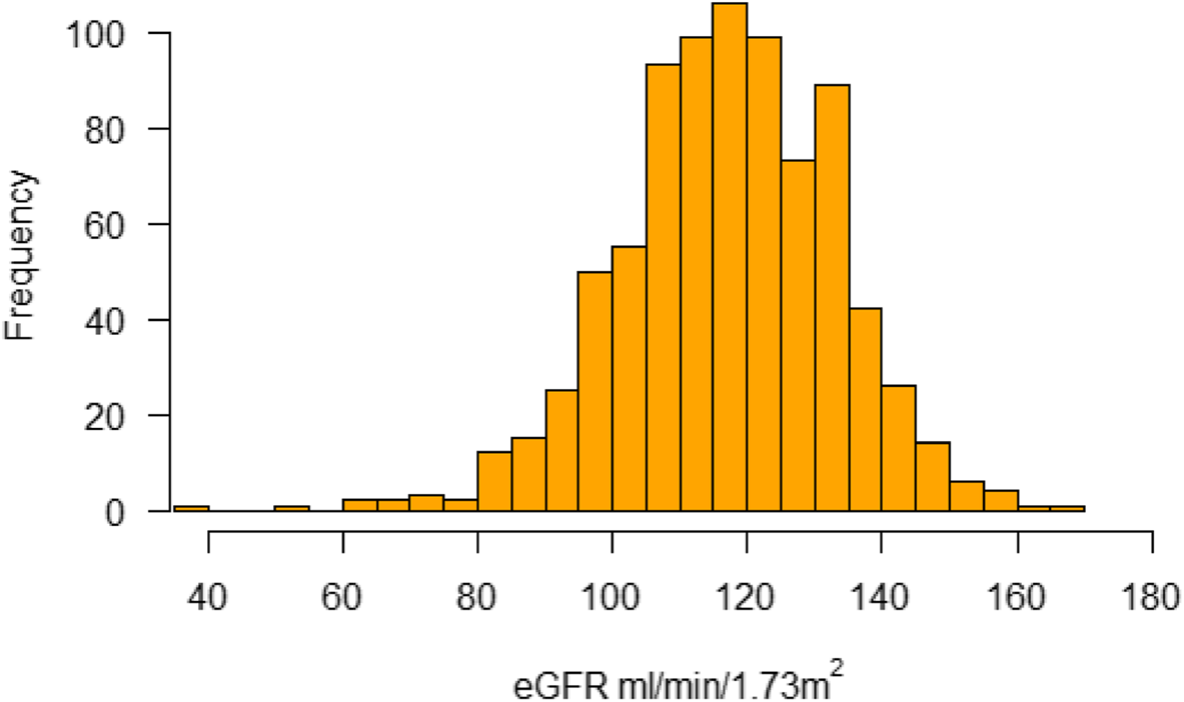
Histogram of eGFR distribution in the sample population

The mean eGFR was 117.1 ± 16.0 ml/min per 1.73m^2^. We observed a lower eGFR with increasing age, increasing BMI, for agricultural workers, for those with fewer years of schooling (≤5 years), for higher income, non-regular meat-eaters (those eating meat 0 days per week) with decreasing fat-free mass, and HIV seropositivity. We observed small site and sex-specific differences in mean eGFR; the mean was 117.6(±14.9) for urban participants, and 116.9(±16.5) for rural participants and 116.8 (±16.7), 117.3 (±15.6) for males and females, respectively. Figure 3 shows the eGFR distribution in the sample population.

The prevalence of eGFR< 60 in the study population was 0.2% (95%CI 0.1, 0.9), and the prevalence of eGFR< 90 was 5% (95% confidence interval (95%CI) = 3.2, 6.3). We observed small differences in reduced eGFR (< 90) prevalence by site, with 3% (1.4, 6 .7) in (urban) Area 25, versus 5% (3.6, 7.8) in (rural) Bonje (Table 1).

### Risk factors for reduced eGFR and eGFR< 90

We first conducted linear and logistic regression models which included all potential risk factors and confounders; however, due to the small number of participants with an eGFR < 90 (*n* = 38), we also repeated the models minimally adjusted for key risk factors and confounders to test for inflation of relative risk due to sparse data. There was little difference between the findings from the fully adjusted and minimally adjusted models, therefore we report results from the fully adjusted models only. Table 2 shows linear and logistic regression models mutually adjusted for age, sex and location (models 1 and 3), and fully adjusted for all risk factor variables (age, sex, location, BMI, FFM, education, occupational status, lifestyle choices, income and non-regular meat-eaters (models 2 and 4). Age was analysed as a continuous variable (in 10-year increments) to estimate overall age-related associations with continuous eGFR in the sample population. HIV status was not included in further analyses due to the small proportion of seropositive participants, and the large proportion of participant with unknown HIV status. Results showed no marked differences between eGFR and levels of adjustment for risk factors (< 10% change in regression coefficients), thus we report results from the fully adjusted models (2 and 4) only.

**Table 2:** Associations between sociodemographic and anthropometric characteristics and eGFR/eGFR< 90 in participants without diabetes, hypertension, proteinuria, *n* = 821

Variable	Model 1 Minimal adjustment	Model 2 Fully adjusted	Model 3 Minimal adjustment	Model 4Fully adjusted
	eGFR	eGFR	eGFR < 90	eGFR < 90
	Coefficient (95%CI)^a^;	Coefficient (95%CI)^b^;	OR (95%CI)^a^;	OR (95%CI)^b^;
Age ^c^ (per 10-year increase)	−8.73 (−9.37, −8.09)	−8.86 (−9.60, −8.15)	3.21 (2.49, 4.29)	3.59 (2.58, 5.21)
Sex ^d^				
Male	0.02 (−1.62, 1.68)	−0.45 (−2.40, 1.47)	0.76 (0.34, 1.67)	1.44 (0.48, 5.05)
Female	Ref	Ref	Ref	Ref
Area				
Urban (Area 25)	Ref	Ref	Ref	Ref
Rural (Bonje)	3.85 (2.05, 5.64)	3.82 (1.51, 6.32)	0.69 (0.27, 1.92)	1.75 (0.50, 6.30)
Education (years)				
≤ 5	4.51 (1.10, 7.93)	4.29 (0.77, 7.69)	0.55 (0.14, 2.02)	0.64 (0.14, 2.56)
> 5 ≤ 10	1.29 (−0.56, 3.15)	1.31 (− 0.57, 3.18)	0.78 (0.30, 2.03)	0.86 (0.31, 2.39)
> 10	Ref	Ref	Ref	Ref
Occupation				
Agricultural worker	−1.06 (−3.14, 1.01)	− 1.11 (− 3.20, 1.02)	0.44 (0.17, 1.21)	0.42 (0.15, 1.17)
Non-agricultural worker	Ref	Ref	Ref	Ref
Household monthly income (MK) ^e^				
Unknown	−1.86 (−7.60, 3.87)	−2.26 (−8.01, 3.50)	1.39 (0.06, 13.66)	2.21 (0.09, 20.58)
MK 0 ≤ 20,000	Ref	Ref	Ref	Ref
MK > 20,000	−0.34 (−2.16, 1.47)	− 0.01 (− 1.85, 1.87)	1.32 (0.54, 3.23)	1.05 (0.40, 2.78)
BMI (kg/m^2^)				
5 kg/m^2^ increase	−0.99 (−2.8, 0.09)	− 0.94 (− 2.02, 0.17)	2.00 (1.29 3.15)	2.01 (1.27, 3.43)
Fat Free Mass (kg)				
(Per 5 kg increase)	−0.02 (− 0.07, 0.02)	−0.02 (− 0.07, 0.02)	1.00 (0.00, 1.02)	0.99 (0.03, 1.03)
Healthy lifestyle choices				
Non-smoker or alcohol drinker	−1.49 (−3.57, 0.59)	− 1.43 (− 3.50, 0.70)	2.07 (0.72, 6.64)	2.06 (0.68, 7.06)
Smoker and alcohol drinker	Ref	Ref	Ref	Ref
Regular meat-eater				
Yes	−0.40 (−2.32, 1.51)	− 0.33 (− 2.27, 1.65)	1.34 (0.58, 3.39)	1.04 (0.43, 2.72)
No	Ref	Ref	Ref	Ref

Hypertension = systolic bp ≥140 mmHg, or diastolic bp ≥90 mmHg; Diabetes = fasting glucose > = 7 mg/l; Proteinuria = ACR > =30 mg/mmol

^a^minimal adjustment for age, sex and area

^b^all variables mutually adjusted

^c^adjusted for sex and area

^d^adjusted for age and area

^e^Exchange rate (MK to USD) 0.001 at time of questionnaire

In the fully adjusted linear regression model (Table 2, model 2), age was a key risk factor for lower eGFR [regression coefficient (95%CI) = − 8.86 (− 9.60, − 8.15)] per ten-year age increase. Rural residence was independently associated with higher eGFR [3.82 (1.51, 6.32)] despite this site having the higher prevalence of eGFR< 90 (6%). Education level < 5 years was also associated with higher eGFR [4.29 (0.77, 7.69)]. No associations were observed between lower eGFR and sex, or occupational status.

Similarly, in the fully adjusted logistic regression model (model 4), the odds of reduced eGFR< 90 increased with age [OR = 3.59 (2.58, 5.21)] per ten-year increase, and with increasing BMI [OR = 2.01 (1.27, 3.43)] per 5 kg/m^2^ weight increase. No increased risk of eGFR< 90 with sex, study site, or occupational status was observed.

### Urban-rural comparison of eGFR

As CKDu had been found to be a predominantly rural disease in areas where it is endemic, we conducted regression analyses to assess site-specific risk factors. We report linear and logistic regression results from fully adjusted models for Bonje (Table 3, models 1, 2). Due to the small proportion of participants in the eGFR< 90 category in the urban population, we could not conduct logistic regression analyses for Area 25 and thus report fully adjusted linear regression results (model 3). For minimally adjusted models, refer to Additional files 2 and 3, Tables S2 and S3.

**Table 3:** Associations between sociodemographic and anthropometric characteristics and estimated glomerular filtration rate (eGFR) (fully adjusted) after removal of those with hypertension, diabetes and proteinuria, Area 25 (*n* = 243) and Bonje (*n* = 578)

Variable	Bonje	Bonje	Area 25
	Model 1	Model 2	Model 3
	eGFR	eGFR < 90	eGFR
	Coefficient (95%CI);	Coefficient (95%CI)^a^;	Coefficient (95%CI)^a^;
Age			
Per 10-year increase	−9.00 (−9.79, −8.22)	3.09 (2.12, 4.59)	− 8.14 (− 10.61, −6.23)
Sex			
Male	−0.10 (−2.45, 2.23)	1.55 (0.37, 8.28)	− 1.62 (− 5.29, 2.05)
Female	Ref	Ref	Ref
Education (years)			
≤ 5	4.19 (0.39, 7.99)	0.92 (0.17, 4.58)	5.59 (−2.63, 13.82)
> 5 ≤ 10	1.65 (−0.38, 3.69)	1.04 (0.33, 3.40)	−0.62 (−5.49, 4.24)
> 10	Ref	Ref	Ref
Occupation			
Agricultural worker	−1.04 (−3.14, 1.04)	0.45 (0.17, 1.27)	0.99 (−13.76, 15.75)
Non-agricultural worker	Ref	Ref	Ref
Household monthly income (MK)^b^			
Unknown	−4.46 (−11.76, 2.82)	3.18 (0.11, 37.04)	−0.83 (− 11.10, 9.43)
MK 0 ≤ 20,000	Ref	Ref	Ref
MK > 20,000	0.35 (−1.67, 2.37)	1.02 (0.37, 2.73)	−1.73 (−6.45, 2.98)
BMI (kg/m^2^)			
5 kg/m^2^ increase	−0.62 (−1.97, 0.73)	1.89 (1.06, 3.81)	−1.58 (−3.54, 0.36)
Fat Free Mass (kg)			
(Per 5 kg increase)	−0.01 (− 0.07, 0.04)	0.92 (0.58, 1.01)	− 0.02 (− 0.11, 0.06)
Healthy lifestyle choices			
Non-smoker or alcohol drinker	−1.40 (−3.95, 1.13)	1.52 (0.43, 5.87)	−1.84 (−5.83, 2.14)
Smoker and alcohol drinker	Ref	Ref	Ref
Regular meat-eater			
Yes	−0.10 (−1.77, 2.77)	1.05 (0.41, 2.93)	−1.95 (−7.38, 3.46)
No	Ref	Ref	Ref

Hypertension = systolic bp ≥140 mmHg, or diastolic bp ≥90 mmHg; Diabetes = fasting glucose > = 7 mg/l; Proteinuria = ACR > =30 mg/mmol

^a^All variables mutually adjusted

^b^Exchange rate (MK to USD) 0.001 at time of questionnaire

Within each site, increasing age was associated with lower eGFR, and risk of reduced eGFR (models 1–3). In Bonje, education < 5 years was associated with higher eGFR [4.19 (0.39, 7.99)]. After further stratification by age however, this association was lost, suggesting age was confounding this relationship due to a higher proportion of younger participants in this category having an eGFR> 90 in comparison to the other education-level categories. In logistic regression, increasing BMI was associated with risk of eGFR< 90 (models 1–2). No associations were observed between reduced eGFR and sex or occupational status within either study site.

## Discussion

We report the distribution of eGFR in participants without hypertension, diabetes, or heavy proteinuria, and estimate the prevalence and potential risk factors of reduced eGFR in an urban and rural study population.

The distribution of eGFR across the population sample showed a high proportion of participants with an eGFR > 120 ml/min/1.73 m^2^, suggesting that low eGFR is not generally a health burden in this population. As we observed only two cases of eGFR< 60 we conducted analyses using the cut-off eGFR< 90. Observing so few cases of eGFR< 60 in Malawi was unexpected, as the country has a similar climate (within a 2-4 °C range of endemic regions) and agricultural-led economy to other countries (in Central America and South Asia) where the rural population is at high risk (see Additional file 4, Table S4 for socioeconomic and environmental factor comparison). This ‘negative’ finding is important, as it contradicts current hypotheses that CKDu is endemic in tropical/subtropical countries and supports the contention that the causes of the epidemic(s) are unknown and more evidence is needed [28, 29].

In our study population, lower eGFR and risk of eGFR< 90 was associated with increasing age, and increasing BMI was associated with increased risk of eGFR< 90. These factors are commonly associated with reduced kidney function both with and without diabetes [30, 31], suggesting that the small poportion of partcipants with reduced eGFR is likely due to age-related renal decline.

Contrary to the current literature that CKDu is likely an agricultural disease [2, 32–34], we observed a positive association between rural area and eGFR after adjustment despite the higher crude prevalence observed in this area. Furthermore, we did not observe a high prevalence of reduced eGFR in the working age rural population. In Bonje, rice is the predominant crop, and we assume most agricultural workers in this population work in the neighbouring schemes. However, we observed no increased risk in this occupational group, even though the increased risks observed in Central America and South Asia are for agricultural workers, specifically rice workers [8, 35–37]. This finding poses the question why we are observing inter-regional differences in risk of reduced eGFR in similar occupational groups working in similar climatic conditions, and therefore could count against the current heat hypothesis [28].

Prior to this study and other CKD research in Malawi [18], CKD was not identified as a health burden in this region, however eGFR was not measured using the DEGREE protocol diagnostic definitions. In the Malawian population, it is likely that survival with reduced renal function in this region is poor, which may result in a lower prevalence than would be found in settings with more developed health services. This factor could provide the primary explanation for the low prevalence of CKD and (potential) CKDu observed across the DEGREE sample population.

Our study has some limitations. Firstly, due to the cross-sectional nature of this study, we had one eGFR measure, and therefore could not differentiate acute kidney injury (AKI) from CKD. Therefore - as is commonly experienced in epidemiological studies - we may have misclassified some AKI as reduced eGFR and overestimated reduced eGFR (< 90) prevalence. There is debate surrounding the diagnostic criteria for CKDu which largely surrounds the exclusion of hypertension and proteinuria, both of which are both causes and consequences of advanced CKD (and CKDu). At early stages of the disease, proteinuria is uncommon in CKDu, and hypertension is not present, by definition; however, both are common in other forms of CKD (including diabetic glomerular disease), and at the population level, most disease is likely to be detected at earlier stages. Currently, the exclusion criteria for hypertension when defining CKDu varies by geographical location, and there is not yet an internationally accepted definition of CKDu [1]. For example, in Meso America those with hypertension with target organ damage or a blood pressure of ≥160 mmHg/100 mmHg are excluded from further analyses, whereas in Sri Lanka those receiving hypertension treatment or those with a blood pressure of ≥160 mmHg/100 mmHg over two measurements are excluded [1]. Under the DEGREE protocol, a more conservative hypertension cut-off of ≥140 mmHg/≥ 90 mmHg is used. This cut-off has also been applied to international studies conducted in India, Sri Lanka and Meso America, and results have still shown there is a substantial burden of CKDu in population samples [2–4]. The rationale for the ‘DEGREE definition’ of eGFR< 60 as a proxy for CKDu is therefore that although this may result in misclassification of a few cases of advanced CKDu as non-CKDu, this is likely to be rare, whereas not doing such exclusions would lead to a large overestimation of CKDu prevalence. Secondly, sampling this nested population could have resulted in selection bias, potentially attenuating estimates. Rural participants were easily located and had a 70% response rate. However, Lilongwe had a 45% response rate, due to external employment, which is likely to be casual agricultural labour. As a result of differential response rates between the urban and rural sites, statistical power was limited which therefore did not allow us to classify this study as having a negative result, but possibly a “non-positive” result, which can be seen in the wide confidence intervals for age in both linear and logistic regression models (Tables 2, 3).

Perhaps most crucially, the CKD-EPI equation has not yet been validated for the African population, which is a major limiting factor in these analyses.

The main strengths of the study are the use of a random selection of population-based participants. Furthermore, DEGREE CKDu definitions were used [1], which aim to facilitate international comparisons of CKDu prevalence and help describe risk factors and identify the causes and mechanisms leading to CKDu.

## Conclusion

In conclusion, our findings indicate that in Malawi reduced eGFR consistent with the definition of CKDu is non-existent. eGFR< 90 in the absence of known CKD risk factors was present, but these cases were associated with traditional CKD factors, age, and BMI. Contrary to the current literature that CKDu is a rural disease disproportionately affecting males, we observed a low prevalence of lower eGFR amongst rural males, and no rural-urban or sex-specific associations with lower eGFR or risk of eGFR< 90 were found. These findings suggest that due to the absence of site, occupation, and sex associations, CKD(u) is not present at least to the extent that it has been observed in other tropical or sub-tropical countries in Central America and South Asia.

This research is informative to the CKDu research field, as we did not observe the disease in a region which has a similar agricultural-led economy and climate to CKDu-endemic regions. These analyses are also important in informing the geographical distribution of CKDu suggesting that the disease is localized to specific countries, as opposed to the entire tropical or subtropical region.

### Supplementary information

**Additional file 1 **: **Table S1.** Sociodemographic and anthropometric characteristics of overall study participants (prior to exclusion of population with diabetes, hypertension, and heavy proteinuria).

**Additional file 2 **: **Table S2.** Linear and logistic regression models, showing both minimally and fully adjusted models Bonje (*n* = 578).

**Additional file 3 **: **Table S3.** Linear regression models, showing both minimally and fully adjusted models, Area 25 (*n* = 243).

**Additional file 4 **: **Table S4.** A comparison of socioeconomic and environmental factors between non-endemic Malawi and endemic regions of Central America and South Asia.

## Data Availability

The datasets analysed during the current study are not publicly available due to the sensitive nature of biological measurements and unique identifiers in the dataset.

## Abbreviations

ACR: Albumin: creatinine ratio
AKI: Acute kidney injury
BIA: Bio impedance analysis
BMI: Body mass index
CI: Confidence interval
CKD-EPI equation: The CKD Epidemiology Collaboration equation
CKDu: Chronic kidney disease of unknown cause
DEGREE: The Disadvantaged Populations eGFR Epidemiology study
eGFR: Estimated glomerular filtration rate
FFM: Fat-free mass
HDSS: Health and Demographic Surveillance Site
IDMS: Isotope dilution mass spectrometry
IQR: Interquartile range
LMIC: Low-and-middle-income country
MK: Malawian Kwacha
OR: Odds ratio
SD: Standard deviation
USD: US Dollar
