# Correction: Prevalence and risk factors for chronic kidney disease of unknown cause in Malawi: a cross-sectional analysis in a rural and urban population

**DOI:** 10.1186/s12882-024-03681-0

**Published:** 2024-08-05

**Authors:** Sophie A. Hamilton, Wisdom P. Nakanga, Josephine E. Prynn, Amelia C. Crampin, Daniela Fecht, Paolo Vineis, Ben Caplin, Neil Pearce, Moffat J. Nyirenda

**Affiliations:** 1 Department of Epidemiology and Biostatistics, Imperial College London, London, UK; 2 MRC Centre for Environment and Health, Imperial College London, London, UK; 3 School of Public Health, Imperial College London, London, UK; 4 Malawi Epidemiology and Intervention Research Unit, Lilongwe, Malawi; 5 Institute of Cardiovascular Science, University College London, London, UK; 6 Departments of Infectious Disease Epidemiology, London School of Hygiene and Tropical Medicine, London, UK; 7 Centre for Nephrology, Division of Medicine, University College London, London, UK; 8 Department of Medical Statistics, London School of Hygiene and Tropical Medicine, London, UK; 9 Centre for Global NCDs, London School of Hygiene and Tropical Medicine, London, UK

**Correction to: BMC Nephrology (2020) 21:387** 10.1186/s12882-020-02034-x

Following publication of the original article [1], the authors identified some errors in Abstract, Tables 1, 2 and 3; Fig. 3 and Supplementary files – correcting an error in the calculation of the eGFR in the published version of the paper.

NB: although these corrections mean that the numbers for the eGFR analyses (e.g. eGFR < 60) have changed, the main findings of the paper have not changed.

Also, the authors wanted to have removed the following sentence under the section “urban rural comparison of eGFR”

Due to the small proportion of participants in the eGFR < 90 category in the urban population, we could not conduct logistic regression analyses for Area 25.

## Corrected Abstract

### Background

An epidemic of chronic kidney disease of unknown cause (CKDu) is occurring in rural communities in tropical regions of low-and middle-income countries in South America and India. Little information is available from Southern African countries which have similar climatic and occupational characteristics to CKDu-endemic countries. We investigated whether CKDu is prevalent in Malawi and identified its potential risk factors in this setting.

### Methods

We conducted a cross-sectional study from January–August 2018 collecting bio samples and anthropometric data in two Malawian populations. The sample comprised adults > 18 years (*n* = 821) without diabetes, hypertension, and proteinuria. Estimates of glomerular filtration rate (eGFR) were calculated using the CKD-EPI2009 equation. Linear and logistic regression models were applied with potential risk factors, to estimate risk of reduced eGFR.

### Results

The mean eGFR was 112.3 ± 22.9 ml/min per 1.73m^2^ and the mean participant age was 33.5 ± 12.7 years. The prevalence of eGFR < 60 was 2.1% (95% confidence interval (95% CI) 1.2, 3.2); the prevalence of eGFR < 90 was 16.1% (95% CI = 13.6, 18.7). We observed a higher prevalence of eGFR < 90 in the urban population (21.8% (16.8, 27.5)), versus rural (13.7% (10.9, 16.7)). Age was associated with increased risk of eGFR < 90 [Odds ratio (OR) (95%CI) = 1.79 (1.53, 2.13) per ten-year increment]. Lower risk of eGFR < 90 was observed for rural participants [OR (95%CI) = 0.43 (0.24, 0.79)].

### Conclusions

Reduced kidney function consistent with the definition of CKDu is not common in the areas of Malawi sampled, compared to that observed in other tropical or sub-tropical countries in Central America and South Asia. Reduced eGFR < 90 was related to age and was more common in urban areas. These findings are important as they contradict some current hypothesis that CKDu is endemic across tropical and sub-tropical countries. This study has enabled standardized comparisons of impaired kidney function between and within tropical/subtropical regions of the world and will help form the basis for further etiological research, surveillance strategies, and the implementation and evaluation of interventions.

## Corrected Tables

**Table 1:** Sociodemographic and anthropometric characteristics of study participants without diabetes, hypertension, and heavy proteinuria) *n* = 821

Variable	eGFR		eGFR Categories, *n*(%)^b^		
	*N* = 821		*N* = 17	*N* = 115	*N* = 689
	**N (%)** ^**a**^	**Mean (SD)**	**< 60**	**≥ 60**,** < 90**	**≥ 90**
**Age**					
< 20	60 (7)	129.2 (21.4)	1 (1.7)	1 (1.7)	58 (96.7)
20–29	312 (38)	120.1 (21.5)	4 (1.3)	33 (10.6)	275 (88.1)
30–39	232 (28)	110.9 (20.6)	6 (2.6)	25 (10.8)	201 (86.6)
40–49	130 (16)	102.7 (19.0)	3 (2.3)	27 (20.7)	100 (76.9)
50–59	46 (6)	97.7 (14.2)	0	10 (21.7)	36 (78.3)
60+	41 (5)	83.1 (17.4)	3 (7.3)	19 (46.3)	19 (46.3)
**Sex**					
Female	504 (61)	111.8 (23.6)	14 (2.8)	70 (13.9)	420 (83.3)
Male	317 (39)	113.1 (21.7)	3 (0.95)	45 (14.1)	269 (84.8)
**Area**					
Urban (Area 25)	243 (29)	110.4 (23.9)	7 (2.9)	46 (18.9)	190 (78.1)
Rural (Bonje)	578 (71)	113.1 (22.4)	10 (1.7)	69 (11.9)	499 (86.3)
**Education (years)**					
≤ 5	59 (7)	104.6 (22.9)	1 (1.7)	17 (28.8)	41 (69.5)
> 5 ≤ 10	301 (36)	112.1 (23.4)	8 (2.7)	35 (11.6)	258 (85.7)
> 10	461 (56)	113.4 (22.4)	8 (1.7)	63 (13.7)	390 (84.6)
**Occupation**					
Agricultural worker	383 (47)	109.9 (20.8)	7 (1.8)	48 (12.5)	328 (85.6)
Non-agricultural worker	438 (53)	114.3 (24.4)	10 (2.3)	67 (15.3)	361 (82.4)
**Household monthly income (MK)** ^**c**^					
Unknown	17 (1)	113.2 (32.0)	1 (5.9)	1 (5.9)	15 (88.2)
MK 0 ≤ 20,000	406 (50)	113.7 (22.7)	7 (1.7)	53 (13.1)	346 (85.2)
MK > 20,000	398 (49)	110.8 (22.6)	9 (2.3)	61 (15.3)	328 (82.4)
**Healthy lifestyle choices**					
Non-smoker/never drink alcohol	630 (77)	111.8 (23.3)	16 (2.6)	86 (13.7)	528 (83.8)
Smoker/alcohol drinker	191 (23)	113.7 (21.3)	1 (0.5)	29 (15.2)	161 (84.3)
**Regular meat eater**					
Yes	621 (76)	111.3 (23.5)	14 (2.3)	87 (14.0)	520 (83.7)
No	200 (24)	112.6 (21.0)	3 (1.5)	28 (14.0)	169 (84.5)
**Body mass index (kg/m** ^**2**^ **)**					
Underweight (≤ 18.5)	45 (6)	118.0 (22.9)	0	8 (17.8)	37 (82.2)
Normal (> 18.5 - ≤ 25)	545 (66)	113.9 (22.2)	8 (1.5)	72 (13.2)	465 (85.3)
Overweight (> 25 - ≤30)	177 (22)	108.4 (22.5)	4 (2.3)	25 (14.1)	148 (83.6)
Obese (> 30)	54 (7)	104.3 (27.4)	5 (9.3)	10 (18.5)	39 (72.2)
**Fat-free mass (kg)**					
1st tertile (≤ 37)	124 (15)	112.7 (19.3)	0	17 (13.7)	107 (86.3)
2nd tertile (> 37 - < 45)	354 (43)	111.3 (24.5)	9 (2.5)	57 (16.1)	288 (81.4)
3rd tertile (≥ 45)	343 (42)	111.3 (22.4)	8 (2.3)	41 (11.9)	294 (85.7)
**HIV status**					
Positive	3 (0.4)	109.7 (21.7)	0	1 (33.3)	2 (66.6)
Negative	595 (73)	112.3 (21.8)	11 (1.8)	75 (12.6)	509 (85.5)
Unknown	223 (27)	112.2 (25.8)	6 (2.7)	39 (17.5)	178 (79.8)

apercentage in columns

bpercentage in rows

cExchange rate (MK to USD) 0.001 at time of questionnaire

**Table 2:** Associations between sociodemographic and anthropometric characteristics and eGFR < 90 in participants without diabetes, hypertension, proteinuria, *n* = 821

	Model 1 minimal adjustment	Model 2 full adjustment	Model 3 minimal adjustment	Model 4 full adjustment
	eGFR	eGFR	eGFR < 90	eGFR < 90
Variable	Coefficient (95%CI)^a^	Coefficient (95%CI)^b^	Coefficient (95%CI)^a^	Coefficient (95%CI)^b^
**Age (per 10-year increase)** ^**c**^	-9.07 (-10.17, -7.98)	-9.20 (-10.44, -7.97)	1.77 (1.54, 2.06)	1.79 (1.53, 2.13)
**Sex** ^**d**^				
Male	1.57 (-1.23, 4.38)	0.45 (-2.85, 3.76)	0.85 (0.56, 1.28)	0.90 (0.55, 1.49)
Female	Ref.	Ref.	Ref.	Ref.
**Study area**				
Urban (Area 25)	Ref.	Ref.	Ref	Ref.
Rural (Bonje)	7.39 (4.34, 10.44)	7.47 (3.43, 11.52)	0.37 (0.24, 0.57)	0.43 (0.24, 0.79)
**Education (years)**				
≤ 5	5.27 (-0.53, 11.09)	5.04 (-0.84, 10.93)	1.13 (0.53, 2.34)	1.08 (0.50, 2.27)
> 5 ≤ 10	1.16 (-2.00, 4.33)	1.20 (-1.99, 4.41)	0.87 (0.54, 1.41)	0.87 (0.53, 1.41)
> 10	Ref.	Ref.	Ref.	Ref.
**Occupation**				
Agricultural worker	-1.05 (-4.59, 2.47)	-1.07 (-4.65, 2.51)	0.69 (0.41, 1.21)	0.69 (0.40, 1.21)
Non-agricultural worker	Ref.	Ref.	Ref.	
**Household monthly income (MK)** ^**e**^				
Unknown	-1.86 (-11.57, 7.84)	-2.95 (-12.75, 6.83)	0.73 (0.11, 3.04)	0.80 (0.11, 3.35)
MK 0 ≤ 20,000	0.10 (-2.99, 3.20)	-0.36 (-3.54, 2.80)	1.21 (0.77, 1.94)	1.29 (0.81, 2.11)
MK > 20,000	Ref.	Ref.	Ref.	Ref.
**BMI (kg/m** ^**2**^ **)**				
Per 5 kg increase	-1.64(-3.48, 0.20)	-1.59 (-3.45, 0.27)	1.09 (0.85, 1.39)	1.10 (0.86, 1.42)
**Fat-free mass**				
Per 5 kg increase	-0.02 (-0.10, 0.05)	-0.01 (-0.10, 0.06)	0.99 (0.93, 1.01)	0.99 (0.00, 1.01)
**Healthy lifestyle choices**				
Non-smoker or alcohol drinker	-2.43 (-5.97, 1.10)	-2.34 (-5.92, 1.22)	1.02 (0.62, 1.73)	1.00 (0.60, 1.71)
Smoker and alcohol drinker	Ref.	Ref.	Ref.	Ref.
Regular meat eater				
Yes	-0.35 (-2.91, 3.62)	-0.29 (-3.04, 3.63)	0.90 (0.55, 1.44)	0.91 (0.55, 1.48)
No	Ref.	Ref.	Ref.	Ref.

Hypertension = systolic bp ≥ 140 mmHg, or diastolic bp ≥ 90 mmHg; Diabetes = fasting glucose > = 7 mg/l; Proteinuria = ACR > = 30 mg/mmol

^a^minimal adjustment for age, sex and area

^b^all variables mutually adjusted

^c^adjusted for sex and area

^d^adjusted for age and area

^e^Exchange rate (MK to USD) 0.001 at time of questionnaire

**Table 3:** Associations between sociodemographic and anthropometric characteristics and estimated glomerular filtration rate (eGFR) (fully adjusted) after removal of those with hypertension, diabetes and proteinuria, Area 25 (*n* = 243) and Bonje (*n* = 578)

	Bonje	Bonje	Area 25	Area 25
	Model 1	Model 2	Model 3	Model 4
Variable	eGFR	eGFR group (< 90)	eGFR	eGFR group (< 90)
	Coefficient (95%CI)^a^	Coefficient (95%CI)^b^	Coefficient (95%CI)^a^	Coefficient (95%CI)^b^
**Age (per 10-year increase)** ^**c**^	-9.42 (-10.71, -8.14)	1.81 (1.50, 2.22)	-7.99 (-11.48, -4.50)	1.73 (1.22, 2.51)
**Sex** ^**d**^				
Male	0.58 (-3.25, 4.43)	1.05 (0.53, 2.21)	-0.76 (-7.46, 5.93)	0.81 (0.37, 1.72)
Female	Ref.	Ref.	Ref.	Ref.
**Education (years)**				
≤ 5	4.34 (-1.89, 10.58)	1.16 (0.46, 2.81)	8.97 (-6.02, 23.97)	0.70 (0.12, 3.05)
> 5 ≤ 10	1.88 (-1.45, 5.23)	0.66 (0.37, 1.20)	-3.05 (-11.92, 5.82)	1.89 (0.78, 4.45)
> 10	Ref.	Ref.	Ref.	Ref.
**Occupation**				
Agricultural worker	-1.03 (-4.47, 2.40)	0.68 (0.38, 1.23)	5.81 (-21.09, 32.71)	0.68 (0.03, 8.22)
Non-agricultural worker	Ref.	Ref.	Ref.	Ref.
**Household monthly income (MK)** ^**e**^				
Unknown	-8.33 (-20.50, 3.82)	1.08 (0.05, 7.34)	3.90 (-13.64, 21.44)	0.89 (0.04, 5.88)
MK 0 ≤ 20,000	-1.07 (-4.39, 2.25)	1.28 (0.74, 2.28)	2.98 (-5.62, 11.59)	1.25 (0.45, 3.20)
MK > 20,000	Ref.	Ref.	Ref.	Ref.
**BMI (kg/m** ^**2**^ **)**				
Per 5 kg increase	-1.46 (-3.69, 0.75)	1.33 (0.94, 1.93)	-2.03 (-5.59, 1.53)	0.93 (0.64, 1.35)
**Fat-free mass**				
Per 5 kg increase	-0.02 (-0.11, 0.07)	0.98 (0.00, 1.01)	0.01 (-0.16, 0.16)	0.99 (0.00, 1.02)
**Healthy lifestyle choices**				
Non-smoker or alcohol drinker	-2.37 (-6.55, 1.80)	1.00 (0.50, 2.05)	-3.13 (-10.41, 4.13)	1.05 (0.16, 1.57)
Smoker and alcohol drinker	Ref.	Ref.	Ref.	Ref.
**Regular meat eater**				
Yes	-0.13 (-3.57, 3.30)	1.06 (0.60, 1.86)	-3.31 (-6.56, 13.20)	0.55 (0.47, 2.54)
No	Ref.	Ref.	Ref.	Ref.

Hypertension = systolic bp ≥ 140 mmHg, or diastolic bp ≥ 90 mmHg; Diabetes = fasting glucose > = 7 mg/l; Proteinuria = ACR > = 30 mg/mmol

^a^minimal adjustment for age and sex

^b^all variables mutually adjusted

^c^adjusted for sex and area

^d^adjusted for age and area

^e^Exchange rate (MK to USD) 0.001 at time of questionnaire

**Fig. 3:**
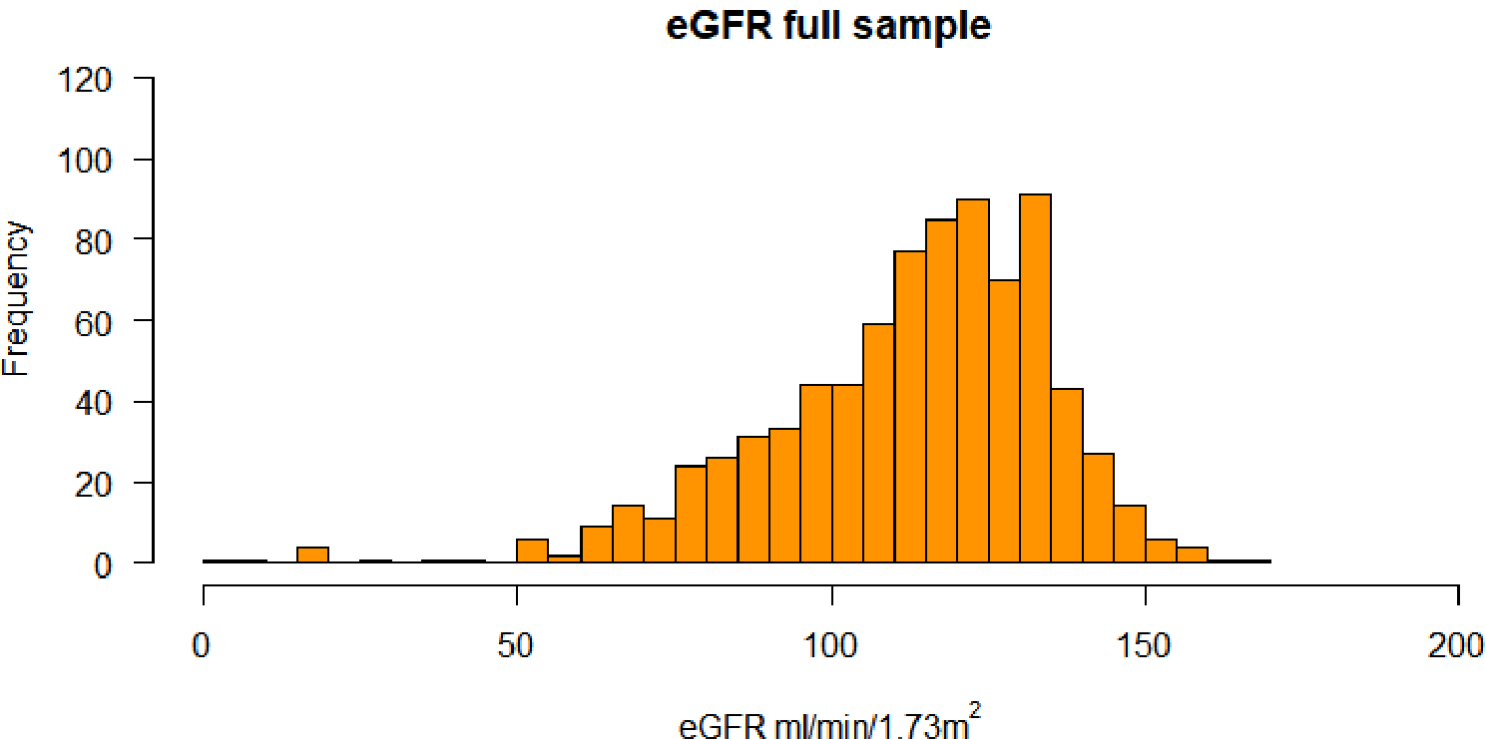
Histogram of eGFR distribution in the sample population

## Supplementary files

**Table S1:** Sociodemographic and anthropometric characteristics of overall study participants (prior to exclusion of population with diabetes, hypertension and heavy proteinuria)

			eGFR categories, *n*(%)^b^			
Variable	eGFR *n* = 1076		*n* = 36	*n* = 167	*n* = 873	
	n(%)^a^	Mean (SD)	< 60	>=60,<90	>=90	
Age (years)						
< 20	66 (6)	129.8(22.3)	1 (2)	2 (3)	63 (96)	
20–29	362 (34)	120.2(21.8)	6 (2)	36 (9)	320 (88)	
30–39	280 (26)	110.6(20.7)	8 (3)	32 (11)	240 (86)	
40–49	185 (17)	101.5 (21.2)	7 (4)	36 (19)	142 (77)	
50–59	79 (7)	97.1(15.1)	2 (3)	16 (20)	61 (77)	
60+	104 (10)	82.9(19.8)	12 (12)	45 (43)	47 (45)	
Sex						
Female	654 (61)	109.3(24.8)	27 (4)	96 (15)	531(81)	
Male	422 (39)	110.5(23.2)	9 (2)	71 (17)	342 (81)	
Education (no of years)						
≤ 5	108 (10)	96.8.(24.3)	7 (6)	31 (29)	70 (65)	
> 5 ≤ 10	399 (37)	110.1(23.7)	15 (4)	48 (12)	336 (84)	
> 10	569 (53)	112.0(23.9)	14 (3)	88 (15)	467 (82)	
Occupation						
Office worker	245 (23)	115.0(25.2)	6 (2)	29 (12)	210 (86)	
Self-employed	133 (12)	109.2(23.1)	5 (4)	22 (17)	106 (79)	
Farmer	493 (46)	107.2(23.0)	18 (3)	75 (15)	400 (81)	
Fisherman	18 (2)	99.1(19.2)	1 (6)	3 (16)	14 (78)	
Unemployed	59 (6)	112.4(23.2)	1 (1)	12 (20)	46 (79)	
Unpaid/domestic worker	113 (11)	114.2(26.0)	3 (3)	18 (16)	92 (81)	
Retired	12 (1)	79.4(19.7)	2 (17)	7(58)	3 (25)	
Refused	3 (0.3)	96.7(26.1)	0	1 (33)	2 (67)	
Household monthly income (MK) ^c^						
< 5000	145 (14)	113.5(26.4)	5 (3)	22 (15)	117 (81)	
> 5,000 < 10,000	177 (16)	110.4(24.4)	5 (3)	23 (13)	149 (84)	
> 10,000 < 20,000	193 (18)	110.5(24.1)	6 (3)	28 (15)	159 (83)	
> 20,000 < 40,000	219 (20)	111.3(22.3)	7 (3)	28 (13)	184 (84)	
> 40,000	321 (30)	106.3(23.5)	11 (3)	63 (20)	247 (77)	
Don’t know	17 (2)	107.4(35.1)	2 (12)	2 (12)	13 (76)	
Refused	4 (0.4)	111.6(26.2)	0	1(25)	3 (75)	
Area						
Urban (Area 25)	326 (30)	108.5(23.9)	11 (3)	63 (19)	252 (77)	
Rural (Bonje)	750 (70)	110.3(24.3)	25(3)	104 (14)	621 (83)	
Healthy lifestyle choices						
Non-smoker/ alcohol drinker	820 (77)	109.5(24.5)	31 (4)	127 (15)	662 (81)	
Smoker/ alcohol drinker	256 (24)	114.8(16.1)	5 (2)	40 (16)	211 (83)	
Regular meat-eater						
Yes	247 (23)	109.9(24.9)	6 (2)	36 (14)	205 (83)	
No	829 (77)	109.2(22.0)	30 (2)	131 (16)	668 (81)	
Body mass index (kg/m^2^)						
Underweight (≤ 18.5)	55 (5)	115.5(25.1)	1(2)	12 (22)	42 (76)	
Normal (> 18.5 - ≤ 25)	677 (63)	111.8(23.80)	19 (3)	95 (14)	563 (83)	
Overweight (> 25 - ≤30)	242 (23)	106.2(22.9)	8 (3)	39 (16)	195 (81)	
Obese (> 30)	102 (10)	101.3(26.9)	8 (8)	21 (21)	73 (72)	
Fat-free mass (kg)						
1st tertile (≤ 37)	171 (16)	106.5(23.4)	7 (4)	31 (18)	133 (78)	
2nd tertile (> 37 - <45)	442 (41)	109.4(25.3)	15 (4)	73 (17)	354 (80)	
3rd tertile (≥ 45)	463 (43)	111.3(23.3)	14 (3)	63 (14)	386 (83)	

^a^ Percentages in columns; ^b^ percentages in rows; Hypertension = systolic bp ≥ 140 mm Hg, or diastolic bp ≥ 90 mm Hg; Diabetes = fasting glucose > = 7 mg/l; Proteinuria = ACR > = 30 mg/mmol; ^c^ Exchange rate (MK to USD) 0.001 at time of questionnare

**Table S2:** Linear and logistic regression models, showing both minimally and fully adjusted models Bonje (*n* = 578)

	Bonje	Bonje	Bonje	Bonje
	Model 1	Model 2	Model 3	Model 4
Variable	eGFR	eGFR	eGFR < 90	eGFR < 90
	Coefficient (95%CI)^a^;	Coefficient (95%CI)^b^;	Coefficient (95%CI)^a^;	Coefficient (95%CI)^b^;
Age^c^				
Per 10-year increase	-9.28 (-10.41, -8.14)	-9.42 (-10.71, -8.14)	1.81 (1.54, 2.16)	1.81 (1.50, 2.22)
Sex^d^				
Male	1.70 (-1.39, 4.81)	0.58 (-3.25, 4.43)	0.90 (0.54, 1.51)	1.05 (0.53, 2.21)
Female	Ref	Ref.	Ref	Ref.
Education (years)				
≤ 5	4.49 (-1.64, 10.64)	4.34 (-1.89, 10.58)	1.21 (0.50, 2.81)	1.16 (0.46, 2.81)
> 5 ≤ 10	1.70 ( -1.60, 5.00)	1.88 (-1.45, 5.23)	0.68 (0.38, 1.22)	0.66 (0.37, 1.20)
> 10	Ref	Ref.	Ref	Ref.
Occupation				
Agricultural worker	-1.01 ( -4.39, 2.37)	-1.03 (-4.47, 2.40)	0.67 (0.39, 1.20)	0.68 (0.38, 1.23)
Non-agricultural worker	Ref	Ref.	Ref	Ref.
Household monthly income (MK)^e^				
Unknown	-7.06 (-18.97, 4.84)	-8.33 (-20.50, 3.82)	0.76 (0.04, 5.27)	1.08 (0.05, 7.34)
MK 0 ≤ 20,000	-0.70 (-3.94, 2.53)	-1.07 (-4.39, 2.25)	1.18 (0.70, 2.06)	1.28 (0.74, 2.28)
MK > 20,000	Ref.	Ref.	Ref.	Ref.
BMI (kg/m^2^)				
5 kg/m^2^ increase	-1.52 ( -3.72, 0.66)	-1.46 (-3.69, 0.75)	1.26 (0.90, 1.75)	1.33 (0.94, 1.93)
Fat Free Mass (kg)				
(Per 5 kg unit increase)	-0.03 (-0.13, 0.05)	-0.02 (-0.11, 0.07)	0.99 (0.00, 1.01)	0.98 (0.00, 1.01)
Healthy lifestyle choices				
Non-smoker or alcohol drinker	-2.52 (-6.62, 1.57)	-2.37 (-6.55, 1.80)	1.06 (0.54, 2.15)	1.00 (0.50, 2.05)
Smoker and alcohol drinker	Ref	Ref.	Ref	Ref.
Regular meat-eater				
Yes	-0.32 ( -3.66, 3.01)	-0.13 (-3.57, 3.30)	1.01 (0.58, 1.72)	1.06 (0.60, 1.86)
No	Ref	Ref.	Ref	Ref.

^a^ Exchange rate (MK to USD) 0.001 at time of questionnaire; Hypertension = systolic bp ≥ 140 mm Hg, or diastolic bp ≥ 90 mm Hg; Diabetes = fasting glucose > = 7 mg/l; Proteinuria = ACR > = 30 mg;

^a^minimal adjustment for age and sex

^b^all variables mutually adjusted

^c^adjusted for sex and

^d^adjusted for age and

^e^Exchange rate (MK to USD) 0.001 at time of questionnaire

**Table S3:** Linear regression models, showing both minimally and fully adjusted models, Area 25 (*n* = 243)

	Area 25	Area 25	Area 25	Area 25
Variable	Model 1	Model 2	Model 3	Model 4
	eGFR	eGFR	eGFR < 90	eGFR < 90
	Coefficient (95%CI)^a^;	Coefficient (95%CI)^b^;	Coefficient (95%CI)^a^;	Coefficient (95%CI)^b^;
Age ^c^				
Per 10-year increase	-8.20 ( -11.08, -5.32)	-7.99 (-11.48, -4.50)	1.64 (1.23, 2.22)	1.73 (1.22, 2.51)
Sex ^d^				
Male	1.20 (-4.87, 7.27)	-0.76 (-7.46, 5.93)	0.77 (0.38, 1.50)	0.81 (0.37, 1.72)
Female	Ref	Ref.	Ref	Ref.
Education (years)				
≤ 5	9.01 (-5.67, 23.71)	8.97 (-6.02, 23.97)	0.71 (0.13, 2.97)	0.70 (0.12, 3.05)
> 5 ≤ 10	-2.13 (-10.79, 6.52)	-3.05 (-11.92, 5.82)	1.81 (0.76, 4.14)	1.89 (0.78, 4.45)
> 10	Ref	Ref.	Ref	Ref.
Occupation				
Agricultural worker	3.23 (-23.29, 29.75)	5.81(-21.09, 32.71)	0.88 (0.04, 9.97)	0.68 (0.03, 8.22)
Non-agricultural worker	Ref	Ref.	Ref	Ref.
Household monthly income (MK)^e^				
Unknown	5.89 (-11.37, 23.16)	3.90 (-13.64, 21.44)	0.68 (0.03, 4.72)	0.89 (0.04, 5.88)
MK 0 ≤ 20,000	3.66 (-4.74, 12.06)	2.98 (-5.62, 11.59)	1.29 (0.47, 3.20)	1.25 (0.45, 3.20)
MK > 20,000	Ref,	Ref.	Ref.	Ref.
BMI (kg/m^2^)				
5 kg/m^2^ increase	-2.07 (-5.56, 1.41)	-2.03 (-5.59, 1.53)	0.93 (0.64, 1.34)	0.93 (0.64, 1.35)
Fat Free Mass (kg)				
(Per 5 kg increase)	0.01 (-0.16, 0.17)	0.01 (-0.16, 0.16)	0.99 (0.00, 1.01)	0.99 (0.00, 1.02)
Healthy lifestyle choices				
Non-smoker or alcohol drinker	-2.64 (-9.81, 4.52)	-3.13 (-10.41, 4.13)	1.04 (0.48, 2.44)	1.05 (0.16, 1.57)
Smoker and alcohol drinker	Ref	Ref.	Ref	Ref.
Regular meat-eater				
Yes	-3.87 (-5.73, 13.47)	-3.31 (-6.56, 13.20)	0.64 (0.20, 1.76)	0.55 (0.47, 2.54)
No	Ref	Ref.	Ref	Ref.

a Exchange rate (MK to USD) 0.001 at time of questionnaire; Hypertension = systolic bp ≥ 140 mm Hg, or diastolic bp ≥ 90 mm Hg; Diabetes = fasting glucose > = 7 mg/l; Proteinuria = ACR > = 30 mg;

aminimal adjustment for age and sex

ball variables mutually adjusted

cadjusted for sex

dadjusted for age

eExchange rate (MK to USD) 0.001 at time of questionnaire
