# Supplementary material for: Prevalence and risk factors for chronic kidney disease of unknown cause in Malawi: a cross-sectional analysis in a rural and urban population
Source: BMC Nephrol. 2020 Sep 7;21:387. doi: 10.1186/s12882-020-02034-x (PMC7487679; doi:10.1186/s12882-020-02034-x)
Supplement: Supplementary file 1 — Additional file 1 : Table S1. Sociodemographic and anthropometric characteristics of overall study participants (prior to exclusion of population with diabetes, hypertension, and heavy proteinuria). [file 12882_2020_2034_MOESM1_ESM.docx]

Table S1. Sociodemographic and anthropometric characteristics of overall study participants (prior to exclusion of population with diabetes, hypertension and heavy proteinuria)

|  |  |  | | |  | eGFR categories, n(%)^b^ | | |
| --- | --- | --- | --- | --- | --- | --- | --- | --- |
| Variable |  | eGFR n= 1076 | | |  | n= 5 | n= 79 | n= 992 |
|  |  | n(%)^a^ |  | Mean (SD) |  | <60 | >=60,<90 | >=90 |
| Age (years) |  |  |  |  |  |  |  |  |
| <20 |  | 66 (6) |  | 133.2 (14.6) |  | 0 | 1 (2) | 65 (99) |
| 20-29 |  | 362 (34) |  | 125.3(12.7) |  | 0 | 3 (1) | 359 (99) |
| 30-39 |  | 280 (26) |  | 115.4 (12.4) |  | 1 (0.4) | 5 (2) | 274 (98) |
| 40-49 |  | 185 (17) |  | 106.9 (12.6) |  | 3 (2) | 5 (3) | 177 (96) |
| 50-59 |  | 79 (7) |  | 100.7 (8.9) |  | 0 | 12 (15) | 67 (85) |
| 60+ |  | 104 (10) |  | 89.0 (11.9) |  | 1 (1) | 53 (51) | 50 (48) |
| Sex |  |  |  |  |  |  |  |  |
| Female |  | 656 (61) |  | 114.9(16.9) |  | 4 (1) | 40 (6) | 610 (93) |
| Male |  | 422 (39) |  | 114.5 (18.0) |  | 1 (0.2) | 39 (9) | 382 (91) |
| Education (no of years) |  |  |  |  |  |  |  |  |
| ≤5 |  | 108 (10) |  | 101.9(18.4) |  | 1 (1) | 24 (22) | 83 (77) |
| >5≤10 |  | 399 (37) |  | 114.6(16.8) |  | 2 (0.2) | 27 (7) | 370 (93) |
| >10 |  | 571 (53) |  | 117.3(16.4) |  | 2 (0.4) | 28 (5) | 539 (94) |
| Occupation |  |  |  |  |  |  |  |  |
| Office worker |  | 245 (23) |  | 120.9(16.7) |  | 1 (0.4) | 8 (3) | 236 (96) |
| Self-employed |  | 133 (12) |  | 114.1(16.2) |  | 1 (1) | 8 (6) | 124 (93) |
| Farmer |  | 493 (46) |  | 111.5 (16.7) |  | 3 (1) | 45 (9) | 445 (90) |
| Fisherman |  | 18 (2) |  | 104.8(13.2) |  | 0 | 2 (11) | 16 (89) |
| Unemployed |  | 59 (6) |  | 117.7(16.4) |  | 0 | 3 (5) | 56 (95) |
| Unpaid/domestic worker |  | 113 (11) |  | 119.4(17.5) |  | 0 | 4 (4) | 109 (95) |
| Retired |  | 12 (1) |  | 86.5 (11.7) |  | 0 | 8 (67) | 4 (33) |
| Refused |  | 3 (0.3) |  | 105.0(21.5) |  | 0 | 1 (33) | 2 (67) |
| Household monthly income (MK) ^c^ |  |  |  |  |  |  |  |  |
| <5000 |  | 145 (14) |  | 117.4(21.0) |  | 0 | 14 (10) | 131 (90) |
| >5,000 <10,000 |  | 177 (16) |  | 114.9 (18.0) |  | 1 (1) | 15 (9) | 161 (91) |
| >10,000 <20,000 |  | 193 (18) |  | 114.8(16.8) |  | 1 (1) | 13 (7) | 179 (93) |
| >20,000 <40,000 |  | 219 (20) |  | 115.4(16.0) |  | 1 (1) | 12 (6) | 206 (94) |
| >40,000 |  | 321 (30) |  | 112.9(15.7) |  | 1 (0.3) | 23 (7) | 297 (93) |
| Don’t know |  | 17 (2) |  | 113.5 (24.2) |  | 1 (6) | 1 (6) | 15 (88) |
| Refused |  | 4 (0.4) |  | 115.9 (24.9) |  | 0 | 1 (25) | 3 (75) |
| Area |  |  |  |  |  |  |  |  |
| Urban (Area 25) |  | 326 (30) |  | 115.6(15.5) |  | 1 (0.3) | 15 (5) | 310 (95) |
| Rural (Bonje) |  | 750 (70) |  | 114.4(18.1) |  | 4 (1) | 64 (9) | 682 (91) |
| Healthy lifestyle choices |  |  |  |  |  |  |  |  |
| Non-smoker/ alcohol drinker |  | 820 (77) |  | 114.6 (17.4) |  | 5 (1) | 60 (7) | 755 (92) |
| Smoker/ alcohol drinker |  | 256 (24) |  | 115.2 (17.2) |  | 0 | 19 (7) | 237 (93) |
| Regular meat-eater |  |  |  |  |  |  |  |  |
| Yes |  | 247 (23) |  | 113.5 (16.4) |  | 0 | 21 (9) | 226 (92) |
| No |  | 829 (77) |  | 115.1(17.6) |  | 5 (1) | 58 (7) | 766 (92) |
| Body mass index (kg/m^2^) |  |  |  |  |  |  |  |  |
| Underweight (≤18.5) |  | 55 (5) |  | 119.3 (20.2) |  | 0 | 6 (11) | 49 (89) |
| Normal (>18.5 - ≤ 25) |  | 677 (63) |  | 116.7(16.9) |  | 1 (0.1) | 40 (6) | 636 (94) |
| Overweight (>25 - ≤30) |  | 242 (23) |  | 111.0(15.8) |  | 3 (1) | 16 (7) | 223 (92) |
| Obese (>30) |  | 102 (10) |  | 108.3(18.5) |  | 1 (1) | 17 (17) | 84 (82) |
| Fat-free mass (kg) |  |  |  |  |  |  |  |  |
| 1^st^ tertile (≤37) |  | 171 (16) |  | 110.2 (18.1) |  | 0 | 26 (15) | 145 (85) |
| 2^nd^ tertile (>37 - <45) |  | 442 (41) |  | 115.0(18.0) |  | 4 (1) | 27 (6) | 411 (93) |
| 3^rd^ tertile (≥45) |  | 463 (43) |  | 116.2 (16.1) |  | 1 (0.2) | 26 (6) | 436 (94) |

^a^ Percentages in columns; ^b^ percentages in rows; Hypertension = systolic bp ≥140 mm Hg, or diastolic bp ≥90 mm Hg; Diabetes = fasting glucose >=7mg/l; Proteinuria = ACR >=30mg/mmol; ^c^ Exchange rate (MK to USD) 0.001 at time of questionnare
