# Supplementary material for: Prevalence and risk factors for chronic kidney disease of unknown cause in Malawi: a cross-sectional analysis in a rural and urban population
Source: BMC Nephrol. 2020 Sep 7;21:387. doi: 10.1186/s12882-020-02034-x (PMC7487679; doi:10.1186/s12882-020-02034-x)
Supplement: Supplementary file 2 — Additional file 2 : Table S2. Linear and logistic regression models, showing both minimally and fully adjusted models Bonje (n = 578). [file 12882_2020_2034_MOESM2_ESM.docx]

Table S2. Linear and logistic regression models, showing both minimally and fully adjusted models Bonje (n=578)

|  |  | Bonje |  | Bonje |  | Bonje |  | Bonje |
| --- | --- | --- | --- | --- | --- | --- | --- | --- |
|  |  | Model 1 |  | Model 2 |  | Model 3 |  | Model 4 |
| Variable |  | eGFR |  | eGFR |  | eGFR <90 |  | eGFR <90 |
|  |  | Coefficient (95%CI); |  | Coefficient (95%CI); |  | Coefficient (95%CI); |  | Coefficient (95%CI); |
| Age ^c^ |  |  |  |  |  |  |  |  |
| Per 10-year increase |  | -8.84(-9.53, -8.14) |  | -9.00 (-9.79, -8.22) |  | 3.04 (2.33, 4.13) |  | 3.09 (2.12, 4.59) |
| Sex ^d^ |  |  |  |  |  |  |  |  |
| Male |  | 0.22 (-1.66, 2.12) |  | -0.10 (-2.45, 2.23) |  | 0.78 (0.32, 1.86) |  | 1.55 (0.37, 8.28) |
| Female |  | Ref |  | Ref |  | Ref |  | Ref |
| Education (years) |  |  |  |  |  |  |  |  |
| ≤5 |  | 4.35 (0.61, 8.09) |  | 4.19 (0.39, 7.99) |  | 0.77 (0.17, 3.33) |  | 0.92 (0.17, 4.58) |
| >5≤10 |  | 1.5(-0.46, 3.55) |  | 1.65 (-0.38, 3.69) |  | 1.00 (0.35, 2.96) |  | 1.04 (0.33, 3.40) |
| >10 |  | Ref |  | Ref |  | Ref |  | Ref |
| Occupation |  |  |  |  |  |  |  |  |
| Agricultural worker |  | -0.99 (-3.05, 1.07) |  | -1.04 (-3.14, 1.04) |  | 0.48 (0.18, 1.31) |  | 0.45 (0.17, 1.27) |
| Non-agricultural worker |  | Ref |  | Ref |  | Ref |  | Ref |
| Household monthly income (MK)^e^ |  |  |  |  |  |  |  |  |
| Unknown |  | -3.86(-11.05, 3.31) |  | -4.46 (-11.76, 2.82) |  | 2.31(0.08, 27.04) |  | 3.18 (0.11, 37.04) |
| MK 0 ≤20,000 |  | Ref |  | Ref |  | Ref |  | Ref |
| MK >20,000 |  | 0.05 (-1.91, 2.03) |  | 0.35 (-1.67, 2.37) |  | 1.18 (0.47, 2.93) |  | 1.02 (0.37, 2.73) |
| BMI (kg/m^2^) |  |  |  |  |  |  |  |  |
| 5kg/m^2^ increase |  | -0.71 (-2.05, 0.62) |  | -0.62 (-1.97, 0.73) |  | 1.76 (1.05, 3.01) |  | 1.89 (1.06, 3.81) |
| Fat Free Mass (kg) |  |  |  |  |  |  |  |  |
| (Per 5 kg unit increase) |  | -0.02 (-0.08, 0.02) |  | -0.01 (-0.07, 0.04) |  | 0.99 (0.00, 1.05) |  | 0.92 (0.58, 1.01) |
| Healthy lifestyle choices |  |  |  |  |  |  |  |  |
| Non-smoker or alcohol drinker |  | -1.53 (-4.03, 0.96) |  | -1.40 (-3.95, 1.13) |  | 1.47 (0.45, 5.13) |  | 1.52 (0.43, 5.87) |
| Smoker and alcohol drinker |  | Ref |  | Ref |  | Ref |  | Ref |
| Regular meat-eater |  |  |  |  |  |  |  |  |
| Yes |  | 0.00 (-2.03, 2.04) |  | -0.10 (-1.77, 2.77) |  | 1.31 (0.53, 3.52) |  | 1.05 (0.41, 2.93) |
| No |  | Ref |  | Ref |  | Ref |  | Ref |

^a^ Exchange rate (MK to USD) 0.001 at time of questionnaire; Hypertension = systolic bp ≥140 mm Hg, or diastolic bp ≥90 mm Hg; Diabetes = fasting glucose >=7mg/l; Proteinuria = ACR >=30mg;
