# Supplementary material for: Prevalence and risk factors for chronic kidney disease of unknown cause in Malawi: a cross-sectional analysis in a rural and urban population
Source: BMC Nephrol. 2020 Sep 7;21:387. doi: 10.1186/s12882-020-02034-x (PMC7487679; doi:10.1186/s12882-020-02034-x)
Supplement: Supplementary file 3 — Additional file 3 : Table S3. Linear regression models, showing both minimally and fully adjusted models, Area 25 (n = 243). [file 12882_2020_2034_MOESM3_ESM.docx]

Table S3. Linear regression models, showing both minimally and fully adjusted models, Area

25 (n=243)

|  |  | Area 25 |  | Area 25 |
| --- | --- | --- | --- | --- |
| Variable |  | Model 1 |  | Model 2 |
|  |  | eGFR |  | eGFR |
|  |  | Coefficient (95%CI); |  | Coefficient (95%CI); |
| Age ^c^ |  |  |  |  |
| Per 10-year increase |  | -8.26 (-9.91, -6.67) |  | -8.14 (-10.61, -6.23) |
| Sex ^d^ |  |  |  |  |
| Male |  | -0.49 (-3.97, 2.98) |  | -1.62 (-5.29, 2.05) |
| Female |  | Ref |  | Ref |
| Education (years) |  |  |  |  |
| ≤5 |  | 5.53 (-2.55, 13.62) |  | 5.59 (-2.63, 13.82) |
| >5≤10 |  | -0.06 (-4.83, 4.570) |  | -0.62 (-5.49, 4.24) |
| >10 |  | Ref |  | Ref |
| Occupation |  |  |  |  |
| Agricultural worker |  | -0.49(-15.09, 14.10) |  | 0.99 (-13.76, 15.75) |
| Non-agricultural worker |  | Ref |  | Ref |
| Household monthly income (MK)^e^ |  |  |  |  |
| Unknown |  | -0.11 (-10.29, 10.05) |  | -0.83 (-11.10, 9.43) |
| MK 0 ≤20,000 |  | Ref |  | Ref |
| MK >20,000 |  | -2.24 (-6.87, 2.38) |  | -1.73 (-6.45, 2.98) |
| BMI (kg/m^2^) |  |  |  |  |
| 5kg/m^2^ increase |  | -1.61 (-3.53, 0.29) |  | -1.58 (-3.54, 0.36) |
| Fat Free Mass (kg) |  |  |  |  |
| (Per 5 kg increase) |  | -0.02 (-0.11, 0.07) |  | -0.02 (-0.11, 0.06) |
| Healthy lifestyle choices |  |  |  |  |
| Non-smoker or alcohol drinker |  | -1.52(-5.46, 2.42) |  | -1.84 (-5.83, 2.14) |
| Smoker and alcohol drinker |  | Ref |  | Ref |
| Regular meat-eater |  |  |  |  |
| Yes |  | 2.62 (-7.90, 2.66) |  | -1.95 (-7.38, 3.46) |
| No |  | Ref |  | Ref |

^a^ Exchange rate (MK to USD) 0.001 at time of questionnaire; Hypertension = systolic bp ≥140 mm Hg, or diastolic bp ≥90 mm Hg; Diabetes = fasting glucose >=7mg/l; Proteinuria = ACR >=30mg;
