# Supplementary material for: Prevalence and risk factors for chronic kidney disease of unknown cause in Malawi: a cross-sectional analysis in a rural and urban population
Source: BMC Nephrol. 2020 Sep 7;21:387. doi: 10.1186/s12882-020-02034-x (PMC7487679; doi:10.1186/s12882-020-02034-x)
Supplement: Supplementary file 4 — Additional file 4 : Table S4. A comparison of socioeconomic and environmental factors between non-endemic Malawi and endemic regions of Central America and South Asia. [file 12882_2020_2034_MOESM4_ESM.docx]

Table S4. Comparison of socioeconomic and environmental factors between Malawi and endemic regions of Central America and South Asia

| Location | Temperature (^o^C)^a^ | Humidity (%)^a^ | Rainfall (mm)^a^ | Elevation (m) | Typical occupation | Main crop(s) | Predominant diet |
| --- | --- | --- | --- | --- | --- | --- | --- |
| Malawi, Africa | | | | | | | |
| Bonje | Min: 15.1  Max: 27.8  Average: 22.8 | Min: 51.3  Max: 85.6  Average: 69.7 | Min: 13.9  Max: 1825.9  Average: 919.9 | 509m | Subsistence farming | Rice, Maize | Maize |
| Area 25 |  |  |  | 1105m | Mixed | - | Maize |
| El Salvador, Central America | | | | | | | |
| San Luis Talpa | Min: 19.3  Max: 28.5  Average: 24.6 | Min: 64.9  Max: 87.5  Average: 76.9 | Min: 10.6  Max: 587.7  Average: 299.2 | 0-50m | Agriculture | Sugarcane | Beans |
| Apastepeque |  |  |  | >500m | Agriculture | Sugarcane | Maize |
| Ataco |  |  |  | 1650m | Agriculture | Coffee | Maize |
| San Jacinto |  |  |  | 650m | Services | - | Maize |
| Nicaragua, Central America | | | | | | | |
| El Porvenir | Min: 21.9  Max: 29.4  Average: 25.5 | Min: 67.1  Max: 92.3  Average: 85.34 | Min: 167.5  Max: 1605.2  Average: 886.4 | 239m | Agriculture | Coffee | Beans |
| El Limón |  |  |  | 118m | Mining | Banana | Rice |
| Chichigalpa |  |  |  | 79m | Agriculture | Sugarcane | Rice |
| El Roblar |  |  |  | 789m | Agriculture | Coffee | Beans |
| Sri Lanka, South Asia | | | | | | | |
| Mihintale | Min: 20.2  Max: 30.1  Average 27.1 | Min: 70.6  Max: 91.8  Average: 85.9 | Min: 196.6  Max: 1091.2  Average: 643.9 | 118m | Agriculture | Rice | Rice |
| Rambewa |  |  |  | 86m |  |  |  |
